# Characterization and in-vitro Alzheimer’s properties of exopolysaccharide from *Bacillus maritimus* MSM1

**DOI:** 10.1038/s41598-023-38172-z

**Published:** 2023-07-14

**Authors:** Manal S. Selim, Sahar S. Mohamed, Mohsen S. Asker, Abeer Y. Ibrahim, Samah A. El-Newary, Mohamed E. El Awady

**Affiliations:** 1 Microbial Biotechnology Department, Institute of Biotechnology Research, National Research Centre, Giza, 12622 Egypt; 2 Medicinal and Aromatic Plants Research Department, Pharmaceutical and Drug Industries Research Institute, National Research Centre, Giza, 12622 Egypt

**Keywords:** Biochemistry, Biotechnology, Drug discovery, Microbiology

## Abstract

Four bacterial isolates were obtained from marine sediments collected from Sahl Hashish, Hurghada Red Sea, Egypt. This study was designed to search for promising anti-Alzheimer natural polysaccharide; therefore, four isolates were screened for exopolysaccharides (EPSs) production and acetylcholinesterase inhibition. The isolate S16 provided the highest EPS yield (7.51 g/L) and acetylcholinesterase inhibition. It was identified morphologically and genetically using 16S rRNA gene sequence analysis as *Bacillus maritimus*. A Physicochemical analysis of S16 exopolysaccharide (BMEPS) was estimated, which pointed to the presence of uronic acid and sulfate (24.7% and 18.3%, respectively). HPLC analysis indicated that mannuronic acid, glucuronic acid, glucose, and mannose are presented in a molar ratio of 0.8:1.0:2.8:2.3, respectively. Furthermore, FT-IR revealed an abundance of β-configurations. The GPC estimated the average molecular weight (*Mw*) as 4.31 × 10^4^ g/mol. BMEPS inhibited *ACh*E (IC_50_; 691.77 ± 8.65 μg/ ml), *BCh*E (IC_50_; 288.27 ± 10.50 μg/ ml), and tyrosinase (IC_50_; 3.34 ± 0.09, 14.00 ± 0.14, and 22.96 ± 1.23 μg/ ml during incubation durations of 10, 20, and 40 min). It also demonstrated a selective anti-inflammatory action against *COX*-2 rather than *COX*-1. Moreover, BMEPS exhibited antioxidant capabilities as free radical and oxygen reactive species (ROS) scavenger, metal chelator, reductant agent, and lipid peroxidation suppressor. These activities are due to the distinct chemical composition. The findings of this study indicate that BMEPS could be considered as promising anti-disease Alzheimer's (AD) material in an *in-vitro* model, which qualifies it for advanced *in-vivo* studies in the discovery of alternative Alzheimer’s treatment.

The most prevalent organic substance in the world is polysaccharide^1^. Polysaccharides are common biological macromolecules that take part in a wide range of physiological functions in humans. It performs a wide range of biological functions, including controlling immunological function, blood pressure, blood sugar, and circulation of blood^1^. Industrial polysaccharides are frequently derived from plants, animals, algae, and microbes. Microorganisms secrete soluble or insoluble polymers called EPSs^2^. Furthermore, microorganisms are considered highly reproducible structures among all polysaccharide providers and are closely regulated, whereas exopolysaccharides (EPS) structures produced from plant and animal sources are influenced by climatic, environmental, and feed circumstances. Mainly, marine environments constitute a sizable and distinctive setting where various bacterial populations are required for essential functions for the survival of the planet's ecology. On the other hand, EPSs are frequently utilized as viscosifying, stabilizing, gelling, or emulsifying agents in the food industry because of their distinctive physical and rheological qualities^2^. Microbial polysaccharides are incorporated into new targets, such as bioflocculants, bio absorbents, heavy metal removal, and drug delivery agents^3^. Additionally, antitumor, antiviral, immunostimulatory, and anti-inflammatory actions are among the biological effects of EPSs^4^. Among microorganisms, *Bacillus* sap. strains produce many types of EPS such as levan, β-(1,3)-glucan^5^, acidic EPS from marine *B. amyloliquefaciens* 3MS 2017^6^, and acidic EPS from *Bacillus* sp. NRC5^7^. Some *Bacillus* EPSs demonstrated exceptional emulsifying, flocculating, heavy metal removal, or medicinal properties^5^.

AD is a chronic neurodegeneration-related disorder^8^. Currently, about ≃ 50 million people are diagnosed with AD, and by 2050, this figure is anticipated to exceed 130 million^9^. The abnormalities associated with several important physiological operations are to blame for the multidimensional toxicity, which also includes cholinergic toxicity, amyloid burden, metal ion toxicity, tau toxicity, biomolecular damage, oxidative stress, immune outrage, neurovascular toxicity, calcium ion dyshomeostasis, lymphatic dysfunction, mitochondrial dysfunctions, α-synuclein mediated toxicity, synaptic malfunctions, membrane toxicity, apoptosis malfunctions, deterioration in telomerase activity, aberrant post-translational modifications, microbial imbalance and infection, hyperglycemia, endoplasmic reticulum stress, hypercholesterolemia, autophagy malfunctions, genetic risk, and insulin resistance as well as diabetes^10^. In the CNS (central nervous system), under normal conditions, metal ions (Cu^II^, Zn^II^, and Fe^III^) play the role of cofactors for enzymes and deliver mitochondrial, and neuronal functions^11^. On the contrary, Zn^II^, Cu^II^, and Fe^II^ coordinate with Aβ and speed up the amyloid accumulation and formation of metal-dependent plaques*.* Aβ-Cu and Aβ-Fe complexes induce the production of excess reactive intermediate species (RIS). RIS are crucial components to induce oxidative stress and neuro-inflammation^8^. Therefore, discovering metal chelators materials is a promising therapeutic approach. Overproduction of RIS (superoxide radical, hydrogen peroxide, hydroxyl radical, nitric oxide, peroxynitrite, and hypochlorous acid) encourages critical oxidative stress that injures lipids and proteins, leading to neuronal death. AD brain tissues considerably suffer from excessive RIS levels^8^. The redox-active metal ions (Cu^II^ and Fe^III^) capture Aβ peptide, stabilize oligomeric species, and work as a depot to produce excessive RIS^12^. Therefore, oxidative stress is the base of AD advancement and a potential target in AD treatments^10^. The neuronal plasma membrane contains a high amount of polyunsaturated fatty acids that are susceptible to peroxidation by RIS, which induces neurotoxic components like 4-hydroxynonenal^13^. In AD brain tissue, oxidative stress-mediated cholesterol microdomains inhibit the antioxidant vitamin E in the lipid membrane. Based on the previous explanation, oxidative stress is the key step in cellular dysfunctions that are related to AD. In parallel, many polysaccharides obtained from organisms have been proven to have antioxidant capacities, including i) metal ion capture (Cu^II^, Zn^II^, and Fe^III^) capture; ii) inhibition of ROS and RNS production; iii) protection of lipids from peroxidation; and iv) neutralization of free radicals. For example, EPS from *Adansonia digitata*^14^, EPS from Novel *Bacillus* sp. M3^15^, EPS from *paenibacillus lactes* NRC1^16^, EPS from *B. amyloliquefaciens* 3MS 2017^6^, and EPS from *Bacillus* sp. NRC5^7^. On the other hand, cholinergic neurons play a crucial role in a variety of cognitive processes, including memory, attention, response, and the processing of sensory information. The impairment of cholinergic neurons is associated with cholinergic toxicity. Thus, improvement of cholinergic neurotransmission remains the main approach in the symptomatic treatment of cognitive and behavioral disorders in the early stages of AD^10^. When acetylcholinesterase (*ACh*E) is present in the synaptic cleft, acetylcholine hydrolyzes into choline and acetic acid. Choline acetyltransferase (*ChA*T) and *ACh*E enzymes control ACh synthesis and breakdown^10^. The defect in *ChA*T activity or hyperactivity of *ACh*E in AD patients prompts a considered reduction in ACh content at the synaptic cleft in the cortex, hippocampus, and amygdala. Thus, repairing cholinergic neuronal malformations is a target for improving cognitive disorders in AD patients. Accordingly, *ACh*E inhibitors prevent brain ACh hydrolysis, which increases the concentrations of brain ACh and improves the deficiency of brain cholinergic neurotransmission. Many polysaccharides from organisms have an *ACh*E inhibitory effect, such as EPS from *Achromobacter piechaudii* NRC2^17^, EPSs from *Isochrysis galbana* and *Nannochloropsis oculate*^18^, and polysaccharides from mushrooms^19^.

On the other hand, tyrosinase catalysis is the conversion of the amino acid L-tyrosine into the compound L-3,4-dihydroxyphenyl amine (L-DOPA). The crucial neurotransmitters norepinephrine and epinephrine are precursors to the key neurotransmitters L-DOPA and dopamine, respectively. Tyrosinase is activated by 14-3-3 proteins through phosphorylation-dependent binding. Tyrosinase and neurodegenerative disorders like Alzheimer's, Parkinson's, and Huntington's diseases are indirectly related due to the 14-3-3 proteins. Also, tyrosinase can injure neurons by producing dopamine quinones, which can oxidize dopamine's catechol ring to produce the extremely reactive compound dopamine quinone. Oxidation of dopamine inhibits dopamine locomotion, glutamate locomotion, and mitochondrial respiration^20^. Tyrosinase inhibitors are medications that can suppress tyrosinase activity. Several polysaccharides of natural origin showed anti-tyrosinase activity, such as the fruit's pericarp polysaccharides, longan^21^, *Usnea longissima* polysaccharides^22^.

The current study was designed to search for a new bioactive exo-polysaccharide (EPS) from marine sources accompanied by its isolation, identification, and characterization, followed by an in-vitro route examination of this polysaccharide in treating Alzheimer’s risk factors: oxidative stress, inflammation progression, neurotransmitter degradation, and accumulation of amyloid-β.

## Results

### Isolation, Screening, and identification of marine bacteria from marine sediments

Four isolates of marine bacteria, S4, S7, S13, and S16, were evaluated, and they were found to have a relatively high yield of EPSs 6.99, 8.66, 7.22, and 7.51 g/L, respectively (Table 1). The EPSs from four isolates have been investigated for their acetylcholinesterase activity. In a primary screening test, EPSs (S4 and S16) showed to be the highest isolates as acetylcholinesterase inhibitors (42.0 and 52.2%, respectively) in a concentration of 1000 µg/mL (Table 1). The EPSs from four isolates have been investigated for their acetylcholinesterase activity. In a primary screening test, EPSs (S4 and S16) showed to be the highest isolate as acetylcholinesterase inhibitor (42.0 and 52.2%, respectively) in a concentration of 1000 µg/mL (Table 1).

**Table 1:** Production of EPSs and acetylcholinesterase inhibition.

Isolates	EPS production (g/L)	Acetyl cholinesterase inhibition (%)			
		100 ppm	250 ppm	500 ppm	1000 ppm
S4	6.99	20.2	28.6	36.0	42.0
S7	8.66	21.7	29.2	32.4	33.6
S13	7.22	6.5	8.5	11.4	12.0
S16	7.51	23.3	31.5	40.8	52.2

**Figure 1:**
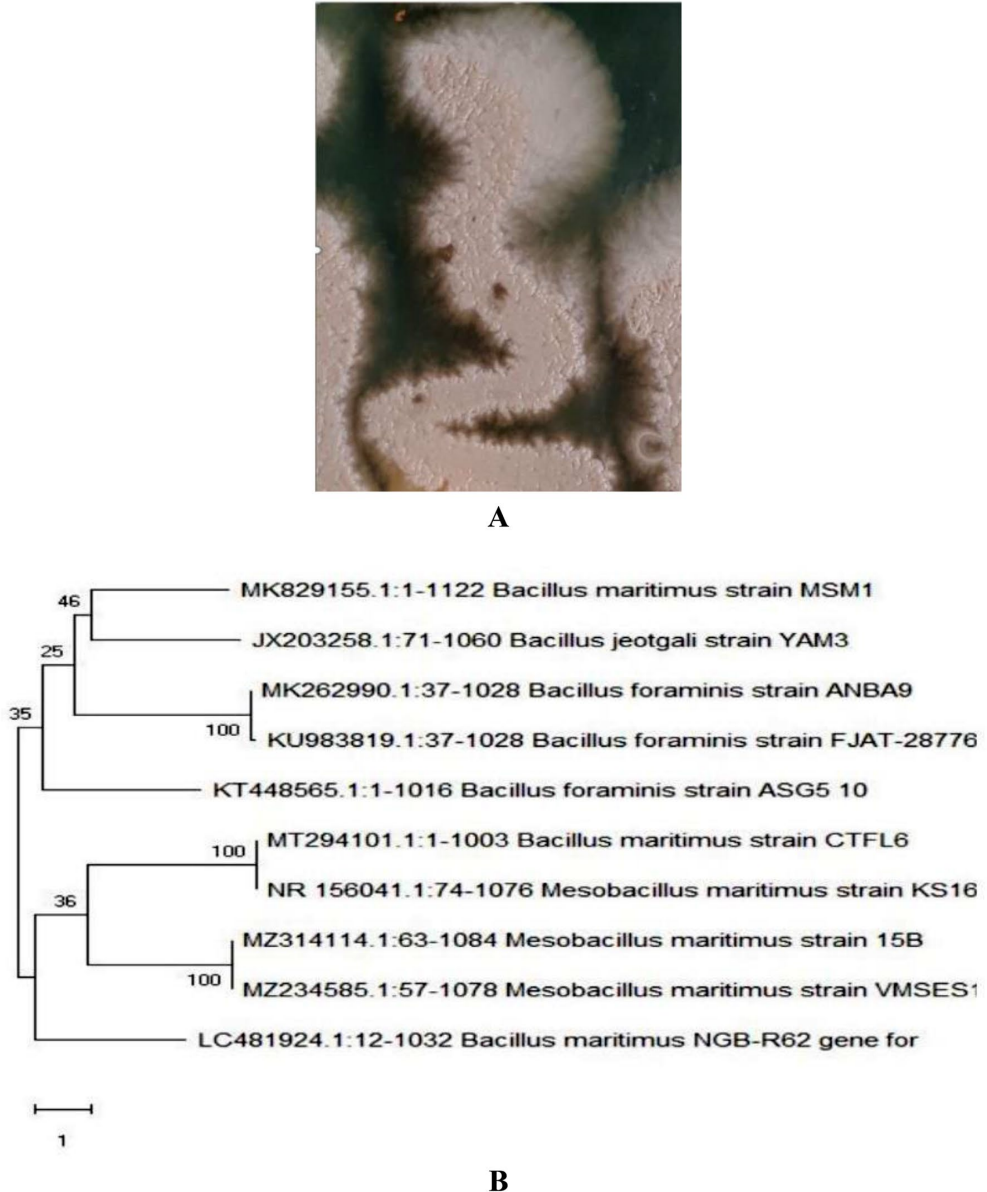
Colony morphology of *Bacillus maritimus* MSM1on solid marine medium (**A**) and Neighbor-joining Phylogenetic tree of strain based on 16S rRNA gene sequences (**B**).

Most bacterial isolate that has high acetyl cholinesterase inhibition activity (S16) was subjected to morphological, physiological, and biochemical characteristics. The colony and morphological characteristics of isolated S16, three days of 37 °C incubation followed the streaking of the pure cultures onto plates. Moreover, the strain formed glistening, slimy, mucoid colonies on agar plates and was capable of producing lengthy strands when expanded using an inoculation loop, exhibiting the typical properties of EPS-producing phenotypes. S16 was identified based on morphological and cultural features, as well as physiological and biochemical studies. Based on the S16 rRNA sequences of strain S16, a phylogenetic tree was created. Bacterial isolate (S16) was primarily identified as gram-positive, endospore-forming, smooth texture, rod shape (Fig. 1A), catalase positive, starch hydrolysis positive, and voges-proskauer (V-P) test, citrate test negative. The phylogenetic analysis of 16S rRNA demonstrated that the promising bacterial isolate (S16) belonged to the gamma subdivision of the Proteobacteria phylum and is closely related to *Bacillus maritimus* MSM1 with accession number MK829155 (Fig. 1B).

### Production, isolation, and chemical analysis of BMEPS

**Figure 2:**
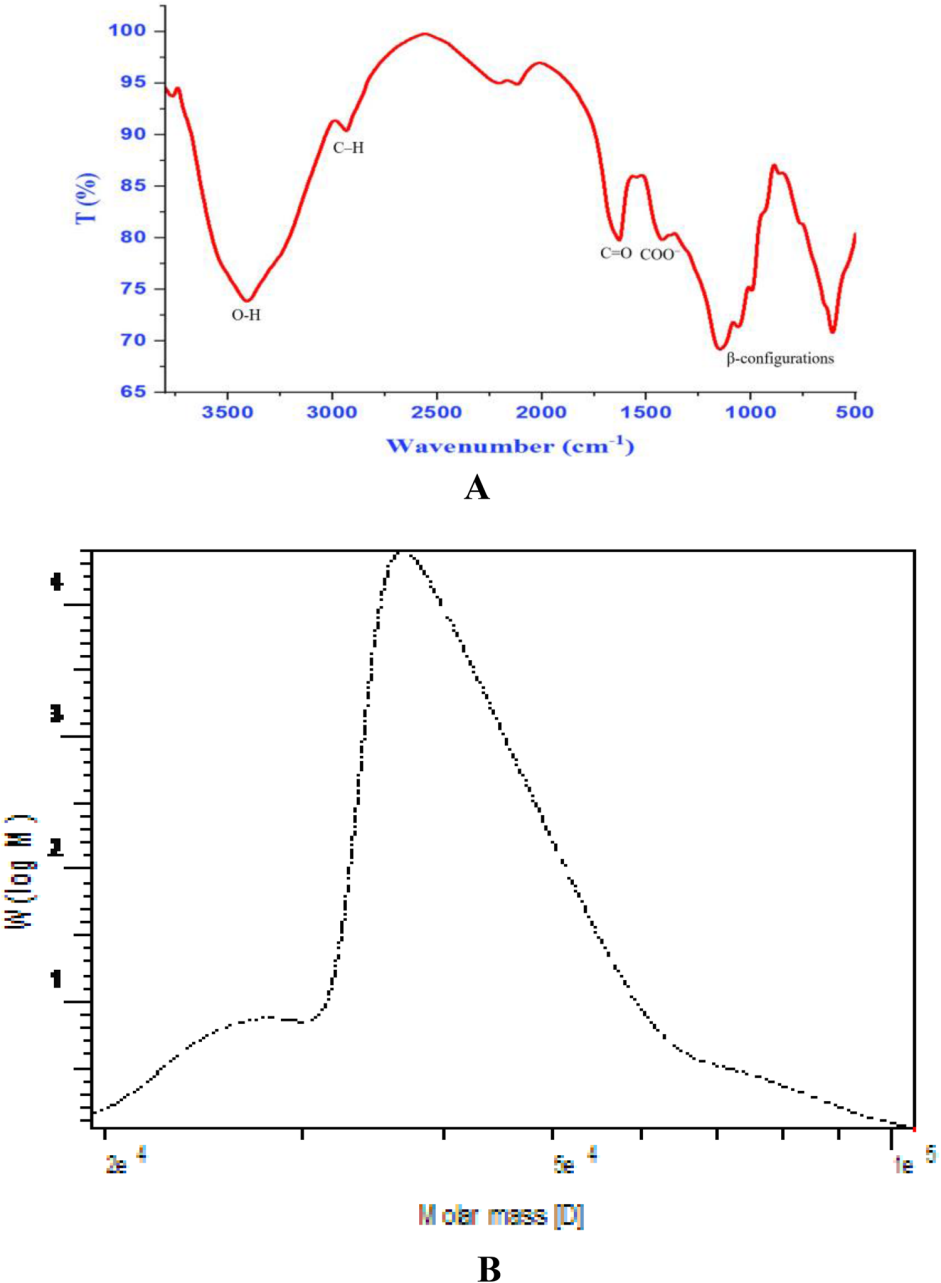
The FTIR spectrum of BMEPS from *Bacillus maritimus* MSM1 (**A**) and molecular weight (**B**).

The high level of EPS was 7.51 g/L when fermented at 37 °C for 3 days, as determined by the phenol–H_2_SO_4_ assay using glucose as a standard. Crude EPS was obtained from the fermented broth by ethanol precipitation and dehydration with acetone and diethyl ether. After centrifugation and the aforementioned ethanol decantation, the crude EPS underwent another precipitation step with cold 100% ethanol. The resulting pellet was then gently heated in a water bath at 50 °C again to achieve complete dissolution. The pellet was then re-dissolved in a minimum amount of deionized water. After that, the pellet was redissolved in a little amount of deionized water, and for the complete dissolving of the pellet, mild heating in a water bath at 50 °C was required once more. The dialyzed solution was fractionally precipitated by 1, 2, and 3 L of cold absolute ethanol after the clear solution's pH was adjusted and dialyzed three times. The yield major fraction obtained by 1 volume of absolute ethanol was dried under vacuum at 40 ºC to obtain BMEPS, which was applied to the following analysis. It showed just one peak at 210 nm in the UV spectra of BMEPS, and there were no peaks between 260 and 290 nm, which showed that there were no proteins or nucleic acids observed in BMEPS. According to an investigation of UV–Vis spectroscopy, the highest absorption was between 200 and 220 nm because of n- and/or-* transitions, which are typical of amine functional groups. Additionally, according to *m*-hydroxydiphenyl colorimetric analysis, BMEPS included 18.3% sulphate and 24.7% uronic acid. The FT-IR spectrum (Fig. 2A) showed a powerful band at 3412.52 cm^−1^, attributed to BMEPS O–H stretching vibration. The 2927.24 cm^−1^ band was caused by C–H stretching vibration. A symmetrical prolonged peak near 1386.72 cm^−1^ showed the existence of COO^−^ groups. The prominent absorption at 1641.52 cm^−1^ was accredited to C = O vibrations. Additionally, distinctive absorptions at 915.22 cm^−1^ suggest the simultaneous presence of β-configurations in BMEPS. The HPLC analysis of the BMEPS chemical structure and comparison to monosaccharide standards were performed. BMEPS was made up of mannuronic acid, glucouronic acid, glucose, and mannose in a molar ratio of 0.8:1.0:2.8:2.3, respectively (Fig. S1, supplementary data). The GPC analysis (Fig. 2B) estimated the average molecular weight (*Mw*) of the BMEPS to be 4.31 × 10^4^ g/mol and the average molecular number (*Mn*) 3.87 × 10^4^ g/mol. The disparity, which is defined as the ratio of *Mw* per *Mn* was equal to 1.1.

### Antioxidant activities

#### Fe^3+^ ions Reductive capacity

**Figure 3:**
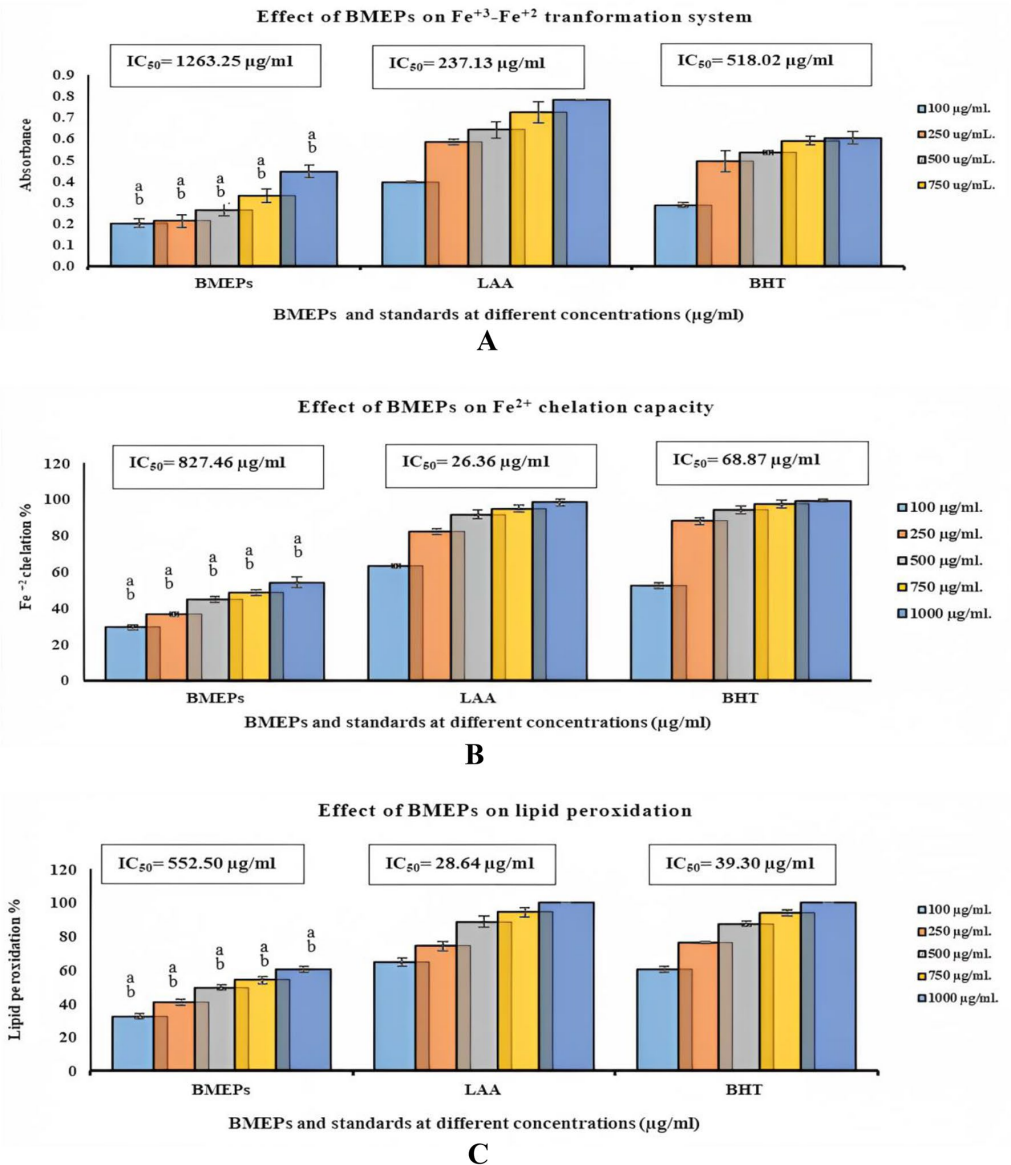
Reduction capability (**A**), metal chelation (**B**), and lipid peroxidation suppression (**C**) effects of BMEPS at different concentrations (100–1000 μg/ ml), compared to reference materials LAA and BHT. (LAA: L-Ascorbic acid and BHT: butylated hydroxytoluene). Data were presented as mean ± SE. ANOVA one-way was used for data analysis (n = 3, *P* ≤ 0.05). Data are followed with small letters; a means significant difference with Ascorbic Acid, b means significant difference with BHT.

Antioxidant compounds are reductants or oxidation inhibitors. Antioxidant substances as reductants can reduce the oxidized Fe^3+^ form into the reduced Fe^2+^ion in the ferricyanide compound. Thereby, the yellow color of Fe^3+^ transforms into numerous green hues, and blue Fe^2+^ is formed based on the reducing power of antioxidant samples. BMEPS showed a weak Fe^3+^ reductive effect, compared to reference materials (Fig. 3A). It showed reducing power represented as absorbance started from 0.200 ± 0.02 at 100 μg/ml and ended with 0.444 ± 0.03 at 1000 μg/ml, in comparison with Ascorbic acid (0.392 ± 0.004 to 0.780 ± 0.003) or BHT (0.287 ± 0.013 to 0.603 ± 0.03) at the same concentrations. IC_50_ of Fe^3+^ was greater than Ascorbic acid or BHT; 1263.26, 237.13, and 518.05 μg/ ml, respectively.

#### Fe^2+^ ions chelation ability

Ferrozine can react with Fe^2+^ ions in the medium and form complexes. Chelator materials will compete with Fe^2+^ to react with Ferrozine if they are present in the reaction medium. Thereby, the ferrozine- Fe^2+^complex red color vanished, indicating that the complex formation was prevented as the assessed chelator material captured Fe^2+^ before ferrozine. The changes in color intensity indicate the co-existing chelator agents^23^.

BMEPS showed moderate Fe^+2^ chelating capacity, compared to reference materials (Fig. 3B). It prevented ferrozine-Fe^+2^ complex formation (29.2% 0 ± 1.51 to 54.04% ± 2.96 at 100 to 1000 μg/ml) compared to LAA (62.99% ± 1.01 to 98.19% ± 1.82) or BHT (52.41% ± 1.59 to 99.09% ± 0.90) at the same concentrations. It could be concluded that IC_50_ of BMEPS (827.46 μg/ml) was greater than LAA or BHT IC_50_ values; 26.36 and 68.88 μg/ml, respectively.

#### Lipid peroxidation inhibition capacity

Lipid peroxidation is a set of chain reactions involving free radicals that can cause a variety of health harm. The unsaturated fatty acids are attacked by ROS, and initiated radical peroxidation chain reactions. When Linoleic acid is incubated with an initiator (Fe^2−^/ H_2_O_2_), it will be oxidized and form hydroperoxides. In the thiocyanate system, ferrous ion is oxidized by linoleate radicals to create ferric ion, which is then measured spectrophotometrically. The antioxidant components restrained the oxidation of ferrous ions leading to prevent the linoleic radical formation in the system^24^.

Concerning the reference materials, BMEPS exhibited potent lipid peroxidation inhibition capability. BMEPS blocked 32.42% ± 1.46 of linoleic acid oxidation in the reaction medium at the lowest concentration (100 μg/ml) in comparison with LAA (64.73% ± 2.20) or BHT (60.31% ± 1.69) at the same concentration. Elevating BMEPS concentration to 1000 µg/ml elevated the inhibition of linoleic acid oxidation to 60.22% ± 1.78, compared to LAA (100% ± 0.0) or BHT (100% ± 0.0) at the same concentrations (Fig. 3C). The 552.50 µg/ml is BMEPS concentration that can inhibit oxidation of 50% of linoleic acid molecules whereas LAA or BHT reference materials represented 28.64 and 39.30 µg/ml, respectively.

#### Free radical scavenging activity

##### DPPH• radical scavenging activity

**Figure 4:**
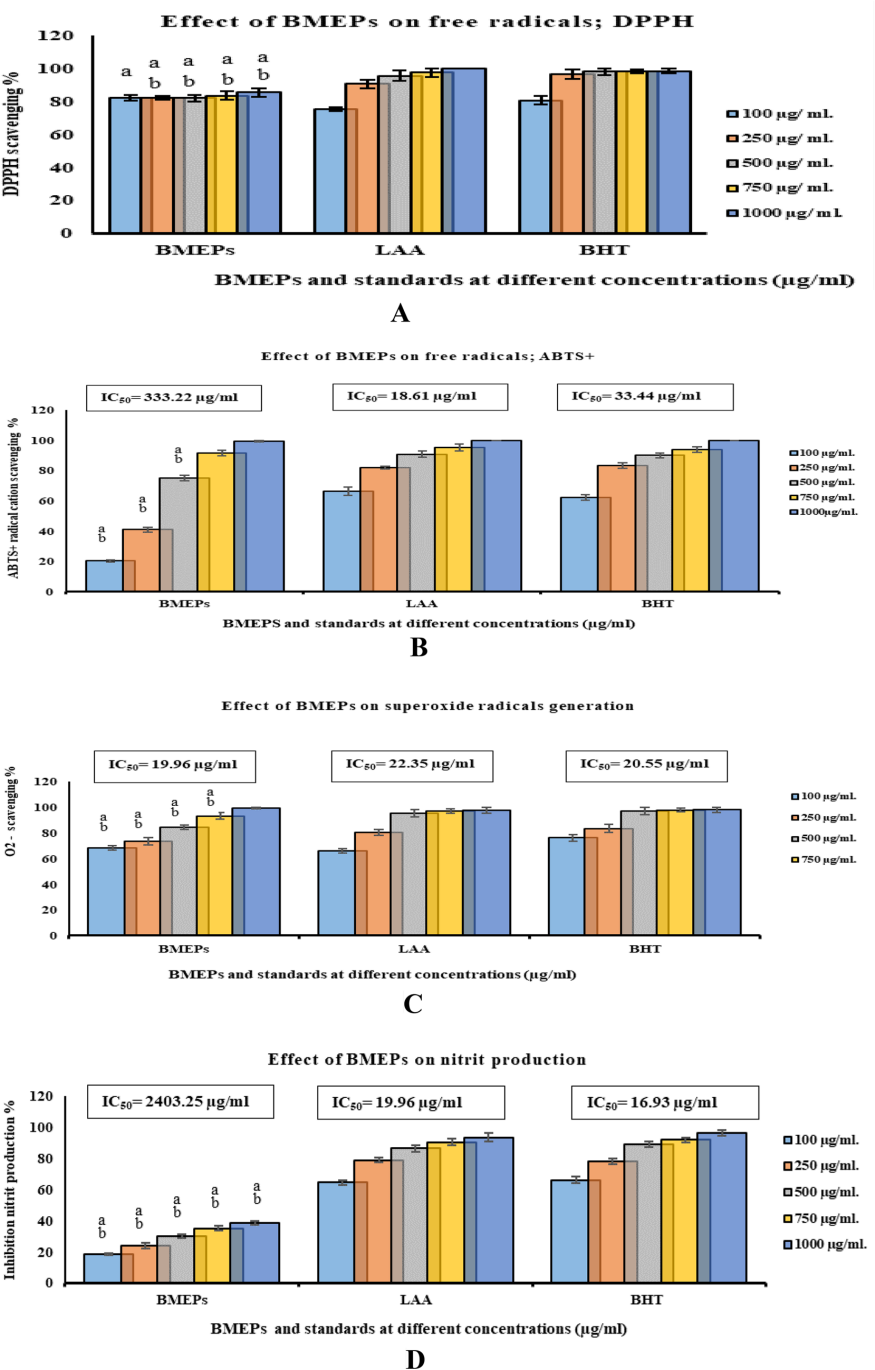
Scavenging capacity of BMEPS; DPPH^•^ (**A**) and ABTS + (**B**), superoxide (**C**), and NO (**D**) at different concentrations (100–1000 μg/ ml), compared to reference materials; LAA and BHT. (NO: nitric oxide radical; LAA: L-Ascorbic acid; BHT: butylated hydroxytoluene; DPPH^.^: 1,1diphenyl-2- picryl-hydrazyl free radical). Data presented as mean ± SE. ANOVA one-way was used for data analysis (n = 3, *P* < *0.05*). Data are followed with small letter; a means significant difference with Ascorbic acid, b means significant difference with BHT.

DPPH• scavenging assay was first suggested in the 1950s originally to discover H-donors in natural materials. DPPH• radical, a chromogen with violet color, is a stable free radical. The DPPH• assay was dependent on the DPPH• reduction in alcohol by a hydrogen-donating antioxidant and formed the non-radical DPPH–H form, yellow-colored diphenyl-picryhydrazine. BMEPS exhibited a potent DPPH• scavenging effect in comparison with reference materials. At the lowest concentration of BMEPS, DPPH• was strongly scavenged (81.67% ± 1.70), compared to LAA (75.17% ± 1.30) or BHT (80.46% ± 2.52) at the same concentrations (Fig. 4A). Elevation of BMEPS concentration to 1000 μg/ml insignificantly increased DPPH• scavenging percentage to 85.37% ± 2.63 in comparison with LAA (100.00% ± 0.00) or BHT (98.32% ± 1.69).

##### ABTS^+^ radical cation scavenging effect

In the decolorization assay, the radical scavenging efficacy of BMEPS was estimated using ABTS^•+^ cation radical, this efficacy was evaluated in comparison with two reference materials; LAA and BHT. ABTS^•+^ cation radical is directly released before the reaction with hydrogen-donating antioxidants and produces blue/green ABTS^•+^ chromophore. ABTS^•+^ may be neutralized either by direct reduction via electron transfers or by radical quenching via hydrogen atom transfer^25^.

BMEPS showed a potent ABTS^+^ cation scavenging ability in a concentration-dependent manner (Fig. 4B). The lowest concentration (100 μg/ml) of BMEPS scavenged low ABTS^+^ scavenging percentage (20.32% ± 0.68) compared to LAA (66.32% ± 2.68) or BHT (62.18% ± 1.81). In accordance, increasing concentration to 1000 µg/ml scavenged more amount of ABTS^+^ cation radical (99.52% ± 0.51) compared to LAA (100.00% ± 0.00) or BHT (100.00% ± 0.00). Insignificant differences were recorded among BMEPS, LAA, and BHT at 750 and 1000 µg/ml. IC_50_ of BMEPs was 333.22 μg/ml whereas LAA or BHT presented 18.61 and 33.44 μg/ml, respectively.

#### ROS scavenging activity

##### O^2-^ radicals scavenging capacity

Superoxide (O^2^), anionic oxygen gas (O_2_), is a main cause of oxidative stress. O_2_·^−^is generated in phenazine methosulphate (PMS)-nicotinamide adenine dinucleotide (NADH) systems by oxidation of NADH and assayed by the reduction of nitro blue tetrazolium (NBT). In this method, O^2^ reduces the yellow dye NBT^2^^+^ to produce the blue formazan which is measured spectrophotometrically at 560 nm. Generally, antioxidants can inhibit blue NBT formation. The decrease in absorbance indicates the consumption of superoxide anion in the reaction mixture meaning the presence of antioxidant material.

BMEPS captured O^2^ radicals in concentrations dependent manner, compared to reference materials (Fig. 4C). In reaction mediums containing BMEPS at consecutive concentrations (100—1000 µg/ ml), 68.45% ± 1.55 to 99.33% ± 0.67 of O^2^ radicals was captured, compared to LAA (66.21% ± 1.79 to 98.11% ± 2.25) and BHT (73.22 ± 2.78 to 98.11% ± 1.89) at the same concentrations. The greatest concentration of BMEPS, LAA, and BHT showed O^2^ scavenging ability was nearly equal. BMEPS concentration to inhibit 50% of generated O^2^ radicals was 19.96 µg/ml, in comparison with LAA and BHT; 22.35 and 20.551 µg/ml, respectively.

##### NO scavenging capacity

Nitric oxide (NO^.^) released from SNP has a powerful NO^+^ which can change the structure and functionality of numerous cellular components. When NO interacts with superoxide to generate the peroxy nitrite anion (. ONOO-), its toxicity rises which is a potentially powerful oxidant that can break down to produce OH and NO_2_^26^.

BMEPS exerted languid action on NO; scavenging percentages were 18.62% ± 0.61 to 38.32% ± 1.19 at concentrations 100 to 1000 μg/ml with concerning LAA (64.39% ± 1.61 to 93.27% ± 2.73) or BHT (65.99% ± 2.00 and 95.88% ± 1.89). IC_50_ of BMEPS was 2403.253 µg/ml compared to LAA and BHT (19.96 and 16.93 µg/ml, Fig. 4D).

### Anti-inflammatory activity

**Figure 5:**
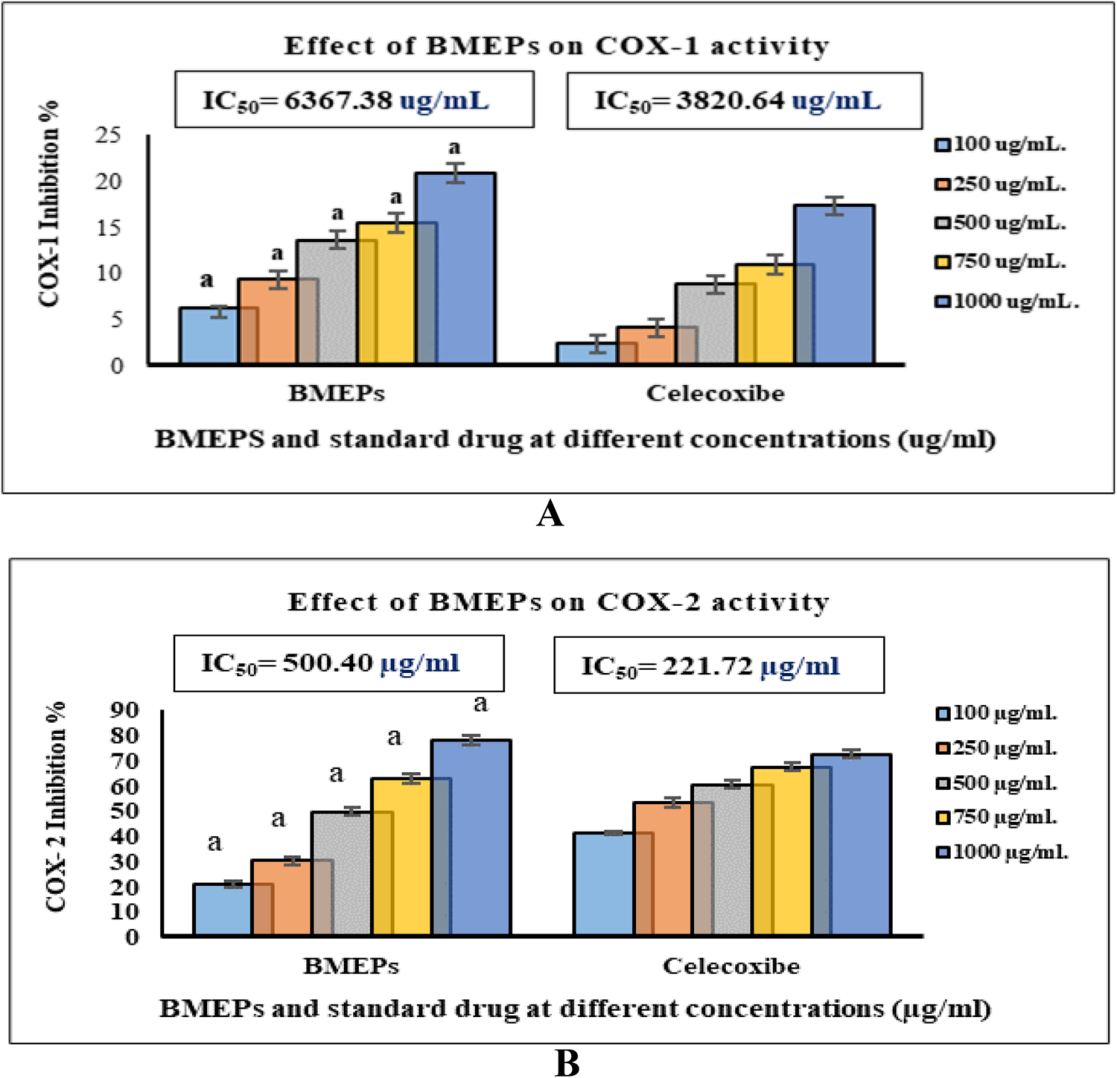
*COX*- 1 (**A**) and *COX*-2 (**B**) inhibition activity of different concentrations (100–1000 μg/mL) of BMEPS and Celecoxib. Data presented as mean ± SE. ANOVA one-way was used for data analysis (n = 3, *p* < *0.05*). Data are followed with a small letter, a means of significant difference with celecoxib.

The BMEPS anti-inflammatory activity was measured by calculating the inhibition percentage of H_2_O_2_ released from leuco-dichlorofluorescein (1-DCF) oxidation in the presence of phenol. Polysaccharide anti-inflammatory effectiveness was examined in comparison to celecoxib as an anti-inflammatory drug with more selectivity towards *COX*-2 than *COX*-1. The repressive effect of BMEPs on *COX*-1 was less than its effect on *COX*-2 (Fig. 5A). *COX*-1 recorded slight inhibition (6.21% ± 0.09 to 20.82% ± 1.00 with BMEPs (100—1000 μg/ mL) compared to inhibition with celecoxib; 2.36% ± 0.77 and 17.24% ± 1.51 at the same concentrations. The IC_50_ of BMEPS (6367.38 μg/ mL) was higher than that of celecoxib (3820.64 μg/ mL) which means this polysaccharide is safer than celecoxib on physiological system.

BMEPS suppressed *COX*-2 activity by about 20.74% ± 1.00, 30.34% ± 1.52, 49.67% ± 1.63, 62.88% ± 1.85, and 78.09% ± 2.00 at 100, 250, 500, 750, and 1000 μg/ mL, correspondingly, compared to celecoxib (Fig. 5B). Accordingly, BMEPS at concentration 500.40 μg/ mL can inhibit 50% of *COX*-2, compared to celecoxib that represented 221.72 μg/ mL.

### Cholinesterase inhibitory effect

**Figure 6:**
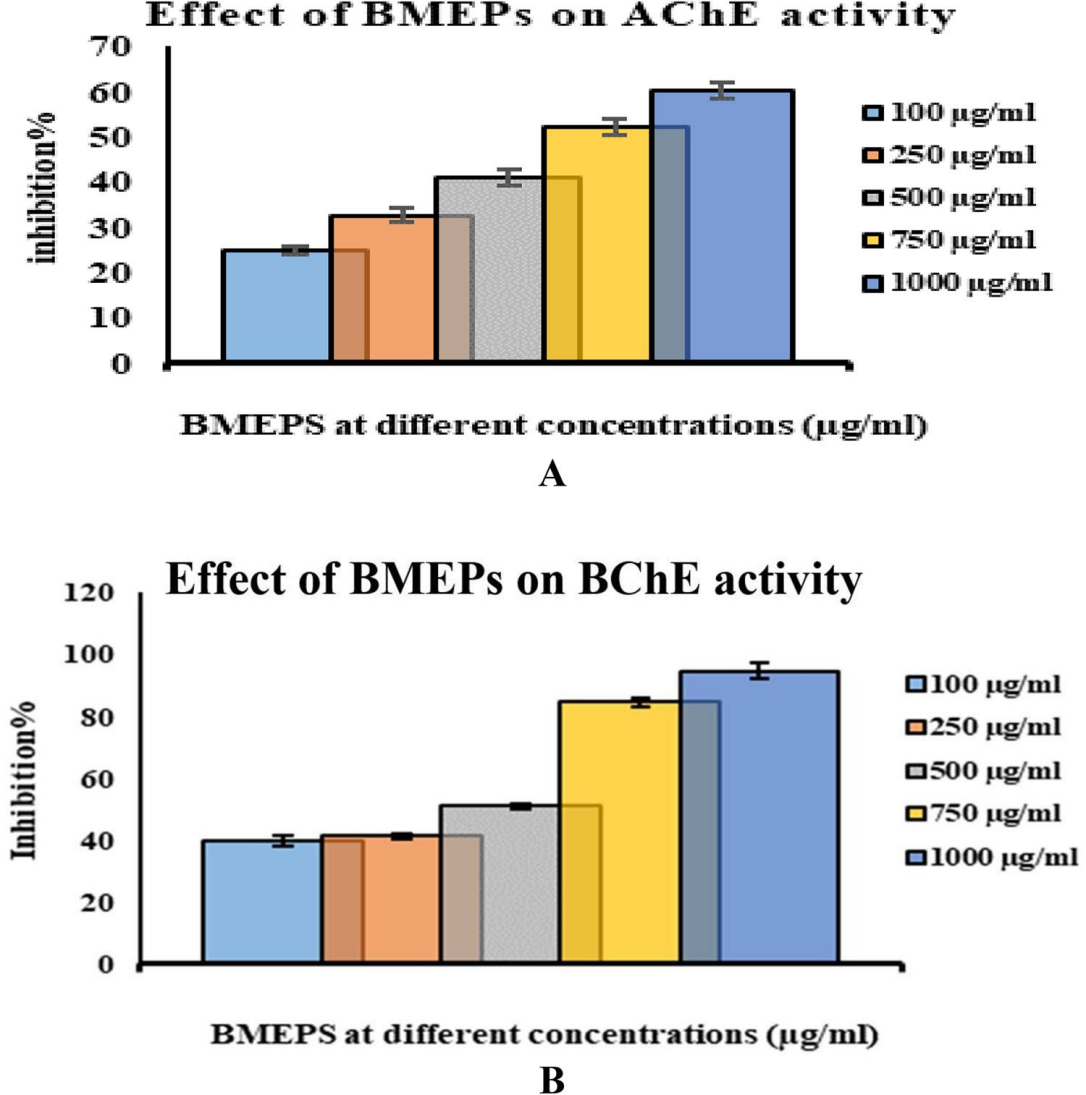
Cholinesterase inhibitory effect of BMEPS at different concentrations (100–1000/ml), *ACh*E (**A**) and *BCh*E (**B**). Data were presented as mean ± SE. ANOVA one-way was used for data analysis (n = 3, *P* ≤ 0.05).

BMEPS had a moderate *ACh*E inhibitory action with concentrations dependent. BMEPS showed *ACh*E inhibition percentages ranging from 24.82% ± 0.88 to 60.15% ± 1.85 at concentrations from 100 to 1000 ug/mL (Fig. 6A). The 691.77 μg/mL is the concentration of BMEPs that blocked 50% of *ACh*E activity. BMEPS exhibited *BCh*E inhibition activity ranging between 39.78% ± 1.73 and 94.62% ± 2.66 at concentrations ranging between 100 and 1000 µg/ml (Fig. 6B). Consequently, 288.27 µg/ml is the concentration that inhibits 50% of an enzyme (IC_50_ of BMEPS).

### Anti-tyrosinase property

**Figure 7:**
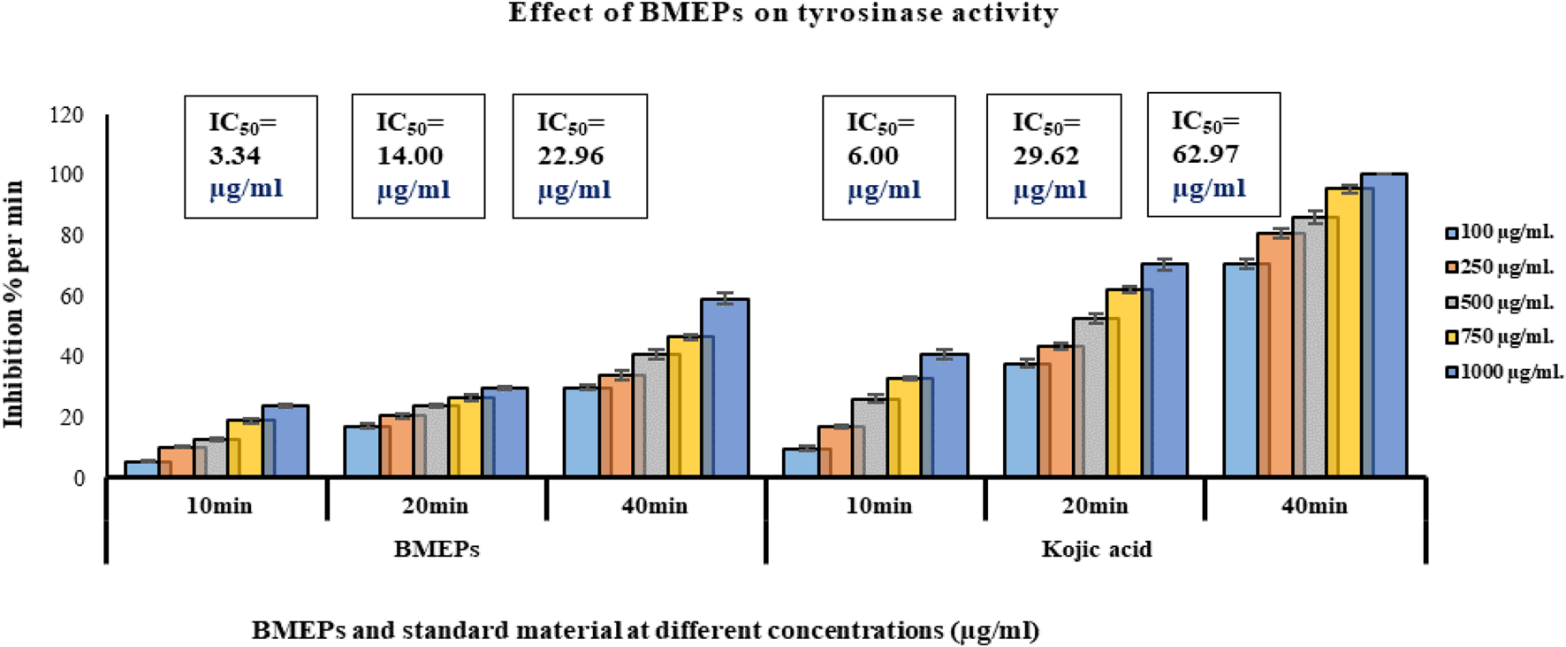
Tyrosinase inhibition activity of different concentrations (100–1000 μg/ ml) of BMEPS and reference materials Kojic acid. Data presented as mean ± SE. ANOVA one-way was used for data analysis (n = 3, *P* < 0.05). Data are followed with small letter; a means significant difference with Kojic acid.

Tyrosinase is an oxidoreductase that contains copper. It speeds up the process of ortho-hydroxylation of monophenols and aerobic oxidation of catechol. The activity of the enzyme was measured by spectrophotometrically measuring the oxidation of 3, 4-dihydroxyphenylalanine (L. DOPA) to the red dopachrome. The anti-tyrosinase activity of BMEPs was determined during three incubation periods (10, 20, and 40 min), and compared to kojic acid as a standard inhibitor. BMEPS showed potent anti-tyrosinase character compared to kojic acid, in concentration and time-dependent action (Fig. 7). Anti-tyrosinase effect of BMEPS was increased by increasing incubation time. The tyrosinase inhibition percentage recorded by BMEPS ranged between 5.18% ± 0.18 to 23.55% ± 0.56 at concentrations 100 to 1000 μg/ml after 10 min incubation compared to kojic acid 9.34% ± 0.66 to 40.35% ± 1.65 at the same concentrations. The inhibition efficacy was increased by increasing time to reach the maximum inhibition after incubation for 40 min; 29.64% ± 0.68 to 58.78% ± 1.85 at concentrations 100 to 1000 μg/ml, compared to kojic acid (70.32% ± 1.68 to 100% ± 0.0) at same concentrations. IC_50_ values of BMEPS at all incubation periods (3.34, 14.00, and 22.96 μg/ml after 10, 20, and 40 min) were less than kojic acid (6.00, 29.62, and 62.97 μg/ml) at the same durations which means the tested polysaccharide is more promising as an anti-tyrosinase inhibitor than kojic acid.

## Discussion

Marine bacteria of different genera are a promising source for the production of several bioactive substances with a variety of biotechnological uses. Many bacterial compounds known as EPSs have a wide range of applications and outstanding physiological activities such as crystallization, emulsification, and antioxidant agents^27^. Four isolates of marine bacteria S4, S7, S13, and S16 were assessed for Eps production and the isolate S16 is found to have a relatively highest EPSs yield and acetylcholinesterase inhibitor efficacy. The promising bacterial isolate (S16) belongs to the gamma subdivision of the Proteobacteria phylum and is closely related to *Bacillus maritimus* MSM1.

The chemical composition of BMEPS was evaluated. Monosaccharide investigation was performed using HPLC and compared to standards. BMEPS was made up of mannuronic acid, glucouronic acid, glucose, and mannose in a molar ratio of 0.8:1.0:2.8:2.3, respectively. The GPC analysis estimated the average molecular weight (*Mw*) of the BMEPS as 4.31 × 10^4^ g/mol and the average molecular number (*Mn*) as 3.87 × 10^4^ g/mol. The disparity, which is defined as the ratio of *Mw* per *Mn* was equal to 1.1. Pentoses, hexoses, or uronic acids are typically structured in repeating units and make up the majority of the EPSs produced by marine bacteria. Due to the fact that location in the gut is determined by chemical composition, the EPSs' composition of glucose and mannose creates an unmixed water layer in the gut, which reduces the absorption of carbohydrates and lipids. Soluble fibers are therefore somewhat effective in the management of diabetes since they can reduce the postprandial rise in blood sugar^28^**.** Most EPSs produced by marine bacteria have OH- and COO- groups, which give them a temporary negative charge and acidic characteristics. The majority of marine EPSs are generally linear, have a range of lengths, and have a mean molecular mass of 1 × 10^5^ to 3 × 10^5^ g/mol^29^. EPS-secreting deep sea hydrothermal bacteria have been reported in recent years to have a high molecular weight, up to 1 × 10^6^ g/mol, frequently acidic, and include uronic acid at a concentration of ~ 30%.

A specific type of brain disease is Alzheimer's disease. It is brought on by harm to the brain's neurons, which are nerve cells. The neurons in the brain are necessary for all human activities, including walking, talking, and thinking. The first neurons to suffer damage in Alzheimer's are those in regions of the brain involved in memory, language, and thought. As a result, memory, language, and thinking issues are frequently the first symptoms. Despite the fact that these symptoms are novel to the individual affected, the brain alterations that cause them are believed to have begun 20 years or more before to symptoms^30^. Aging, genetics, head injuries, vascular illnesses, infections, and environmental variables like oxidative stress, heavy metals, trace metals, and brain inflammation, among others, have all been considered risk factors for Alzheimer's disease (AD). It is currently unknown what causes the pathological alterations in Alzheimer's disease (A, NFTs, and synaptic loss). Several hypotheses were proposed as a cause for AD but two of them are believed to be the main cause: some believe that an impairment in the cholinergic function is a critical risk factor for AD, while others suggest that alteration in amyloid β-protein production and processing is the main initiating factor. However, no widely accepted theory for the pathogenesis of AD exists at this time^31^.

Around 24 million people have been reported to have Alzheimer's disease worldwide as of right now, and by 2050, experts predict that number will have quadrupled. Even though AD is an issue of public health, only two kinds of medications are currently approved to treat AD: cholinesterase enzyme inhibitors (naturally occurring, synthetic, and hybrid analogues), and N-methyl D-aspartate (NMDA) antagonists^32^. Recent research has revealed that natural substances have a neuroprotective effect, which they have long been utilized as therapeutic agents for treating a variety of degenerative disorders. Natural substances have been shown to offer therapeutic potential for AD in both in vitro and in vivo studies, allowing some of them to move into the clinical trial stages. The first natural substance to enter clinical trials for AD was nicotine. Subsequently, additional substances, including as vitamins C, E, and D, attracted increasing interest due to their protective effects against neuroinflammation and oxidative damage. Recently, bryostatin, a macrolide lactone extract from *bryozoan Bugula neritina,* has been evaluated and showed the ability to induce α-secretase activity, reduce Aβ production, and enhance the learning and memory in an AD mice model^33^. Other natural compounds used in folk medicine (traditional Chinese medicine) have shown promising results in the treatment of Alzheimer's disease by working on several mechanisms^34^.

In the present study, the antioxidant capacities of BMEPS from *B. maritimus* MSM1 was evaluated. In general, BMEPS showed good antioxidant features where it; (i) neutralized free radicals (DPPH^.^ and ABTS^+^), (ii) scavenged RS (O^2−^ and NO), (iii) chelated Fe^+2^ ions, and (iv) inhibited lipid peroxidation, in comparison with two reference materials LAA and BHT. In accordance, the antioxidant properties of natural polysaccharides were documented in several studies. Our study was supported by Ibrahim et al.^14^ findings on EPSs from *Adansonia digitate*, El-Newary et al.^6^ acidic EPS from marine *B. amyloliquefaciens* 3MS 2017, acidic EPS produced from *Bacillus* sp. NRC5^7^.

BMEPS antioxidant properties could be discussed depending on its chemical composition. The natural polysaccharides' antioxidant activity is affected by several factors including structural characteristics, the number of active groups (OH, COOH, and SO_4_), sulfate content and binding position, molecular weight linkages, and molecular weight^35^. The antioxidant characteristics of current polysaccharide (PS) could be explained depending on many axes as follows: First axis, the monosaccharides content of PS is associated with antioxidant characteristics, particularly, rhamnose and mannose^36^. The second axis, glycosyl linkages type plays an essential function in the antioxidant properties, specifically, arabinose (1– 4) and mannose (1–2) of the side chain that is strongly linked to the reductive ability, meanwhile glucose (1–6) and arabinose (1– 4) are closely correlated with DPPH radical scavenging activity^37^. The third axis, uronic acids in acidic PS make them potent antioxidants agents^38^ and uronic acids exerted promising free radical scavenging and reductive capacity in the following order; poly-galactouronic acid > glucuronic acid > galactouronic acid^38^. The fourth axis, molecular weight has a strong effect on antioxidant capacities, where low molecular weight polysaccharide is superior to molecules with high masses. PS with low molecular weight has potent reducing power to neutralize the free radicals. Xing et al.^39^ demonstrated that chitosan that has a low molecular weight (9 kDa) exhibited O^2−^ scavenger efficacy better than chitosan with a high molecular weight (760 kDa). Additionally, Rice polysaccharides, with low molecular weights, have promising reducing power, metal chelation, and free radicals scavenging abilities. The fifth axis is attached function groups, sulfated low molecular weight polysaccharides such as *Ulva pertusa* polysaccharides have better antioxidant abilities than the sulfated polysaccharide with high molecular weight PS^40^. Finally, sulfate groups increased the ability of sulfated polysaccharides as free radicals’ scavengers, metal chelators, and inhibition of lipid peroxidation formation^38^. High sulfate-content polysaccharides were more effective than the low ones^41^. Scavenging free radicals of materials is via electron transfer or hydrogen donates from the antioxidant materials to this radical to be a stable form^24^. The PS can donate hydrogen atoms because hydrogen bonds have low dissociation energies. Polysaccharide hydrogen bonds are weak because of the position of the sulfur in the sulfated polysaccharides. Also, sulfate groups of sulfated PS trapped free radicals electrostatically^42^. The sixth axis is pH, the antioxidant abilities of acidic low molecular weight polysaccharides are better than acidic high molecular weight polysaccharides^43^. According to the structure–activity relationship mentioned before, it could be suggested that the antioxidant properties of BMEPS could be associated with its low molecular weight (4.31 × 10^4^ g/mol), uronic acids (24.7%), sulfate (18.3%), monosaccharaides composition; mannuronic acid, glucouronic acid, glucose and mannose in a molar ratio of 0.8:1.0:2.8:2.3, respectively.

This study demonstrated the selective anti-inflammatory ability of BMEPS against *COX*-2 compared to *COX*-1. The outcomes attained were in line with Ibrahim et al.^14^ on *Adansonia digitate* polysaccharide, El-Newary et al.^6^ on acidic EPS obtained from *B. amyloliquefaciens* 3MS 2017, and Mohamed et al.^7^ on acidic EPS from *Bacillus* sp NRC5.

The main factors of inflammatory reaction could be summarized in the following (i) Nitric Oxide (NO) and prostaglandin synthesis, (ii) NF kappa B expression, (iii) reactive oxygen species (ROS), (iv) migration of leukocytes, and finally (v) the pro-inflammatory cytokines i.e. TNF, IL6, and IL1^44^. The anti-inflammatory effect of BMEPS if tested in an in-vivo model could be mentioned through its effect on oxidative stress particularly NO and ROS scavenging ability which was recorded in this study. In the current study, BMEPS exhibited good free radicals scavenging; NRS and ROS, metal chelation, reducing power, and lipid peroxidation inhibition efficacy. BMEPS significantly reduced NO concentration, which in turn reduces and controls inflammation progression in-vivo. Also, BMEPS considerably neutralized superoxide radicals, which in turn, reduces NO toxicity.

Additionally, BMEPS appeared anti-cholinesterase activity against either *ACh*E or *BCh*E. Several polysaccharides have *ACh*E inhibitory action. For example, proteoglucan (PGM) from mushroom^45^, EPSs from the *L. delbrueckii* subsp. *bulgaricus* B3 and *L. plantarum* GD2^46^, EPSs produced from brown macroalgae *Ecklonia radiata*^47^. In agreement, Park et al.^48^ demonstrated that sulfated polysaccharide produced from *Ecklonia cava* exhibited competitive and non-competitive suppressor effects on acetylcholinesterase in PC12 cells induced by H_2_O_2_. In addition, sulfated polysaccharides produced from marine algae such as *E. maxima*, *G. pristoides*, *U. lactuca*, *U. rigida*, and *G. gracilis*, significantly reduced the acetyl cholinesterase activity in treated cells with Zn (50 μM) alone^49,50^.

On the other hand, *ACh*E is significantly affected by metal toxicity as Ze and Cu. In an experiment on fish *Leporinus obtusidens* (piava), exposure to zinc and copper significantly activated *ACh*E^51^. Also, exposure of zebrafish to ZnCl_2_ higher than 1 ppm produced Alzheimer’s disease (AD)-like syndrome including suppression of ACh, decrease locomotor behavior, and impairment in short-term memory due to activation on *ACh*E. Moreover, ZnCl_2_ induces amyloid-β and phosphorylated Tau protein levels in brains^52^. Therefore, capturing excess of these metals from media could inhibit AChE, and increases ACh content in the synaptic area. In this study, BMEPS exhibited metal chelating ability, which could be the reason for it being anticholinergic in *in-vivo* applications. On the other hand, sulfated compounds can react to the anionic site of *ACh*E and reduce its activity^48^ which means that the used sulphated polysaccharide in this study may in part show its inhibitory action on AChE in this way.

The other mechanism that was examined in this study is the tyrosinase pathway. There are many tyrosinase inhibitors, both from synthetic and natural sources. The mechanisms of tyrosinase inhibition include one or more of the following; (i) dopaquinone reduction like ascorbic acid, (ii) o-dopaquinone scavenging as in thio-containing compounds^53^, (iii) Copper chelation that can regulate tyrosinase activity due to the copper structure of tyrosinase as kojic acid^54^ and (iv) H_2_O_2_ scavenging materials, where H_2_O_2_ activated tyrosinase and served as the second substrate^55^. According to the previously presented explanation, we could conclude that the anti-tyrosinase activity of BMEPS is related to being a metal chelator, H_2_O_2_ scavenger, and its content of sulfated groups.

In conclusion, the marine Exo-polysaccharide produced from *Bacillus maritimus* MSM1 was characterized by Physico-chemical analysis using UV, FTIR, HPLC, and GPC analyses. These analyses proved that it is a sulphated low molecular weight polysaccharide with uronic substitution (24.7% and 18.3%, respectively) and the monosaccharides are presented in molar ratio 0.8:1.0:2.8:2.3 for mannuronic acid, glucouronic acid, glucose, and mannose, respectively, with the abundance of β-configurations. The GPC estimated the average molecular weight (*Mw*) as 4.31 × 10^4^ g/mol. BMEPS exhibited promising antioxidant characters and selective inhibitory action against *COX*-2. The proved efficacies support the incorporation of this polysaccharide as a promising alternative drug in treating inflammatory and oxidative stress-related diseases. Additionally, BMEPS shows anti-cholinesterase and anti-tyrosinase activities which are relevant to neurodegenerative diseases. The promising biological features of BMEPS may be attributed to being rich in SO_3_ and COO − groups, β-glycosidic linkage, in addition, its low molecular weight. It is evident from the study that BMEPS can be a candidate in further advanced medical studies as a new natural raw material for Alzheimer’s treatment drugs.

## Materials and methods

### Chemicals

1,1-diphenyl-2-picryl-hydrazyl (DPPH), peroxidase, H_2_O_2_, ABTS (2,2-azino-bis (3-ethylbenz-thiazoline-6-sulfonic acid, polyoxyethylene sorbitan monolaurate (Tween-20), Ascorbic acid (vitamin C), butylated hydroxytoluene (BHT), nicotinamide adenine dinucleotide (NADH), nitroblue tetrazolium salt (NBT), phenazine methosulphate (PMS), sodium nitroprusside (SNP), sulfanilamide, ortho-H_3_PO_4_, naphthylethylene diamine dihydrochloride, diammonium salt, 3-(2-pyridyl)-5,6-bis (4-phenyl-sulfonic acid)-1,2,4-triazine (ferrozine), Ferrous chloride, trichloroacetic acid (TCA), potassium ferricyanide, Leuco-2,7-dichlorofluorescien diacetate, hematin, arachidonic acid, Cyclooxygenases enzymes (*COX*-1 from sheep, EC. 1.14.99.1 for *COX*-2), acetylcholine esterase enzyme, acetylthiocholine iodide, DTNB, butyryl thio-choline Iodide, butyrylcholinesterase enzyme, kojic acid, tyrosinase enzyme, and L-Dopa were purchased from Sigma-Aldrich, USA. Ammonium thiocyanate was purchased from E. Merck. All chemicals and solvents are analytical grades.

### Isolation, screening, and identification of marine bacteria for marine sediments

#### Sampling

Three samples were collected from marine sediment at different locations in Sahl Hashish, Hurghada Red Sea, Egypt.

#### Isolation of bacteria

The collected samples (10 g) were placed in 100 mL of sterile seawater and homogenized by shaking at 200 rpm for 20 min, and a serial dilution was performed^56^. Finally, 50 μL of the supernatant of each dilution was inoculated on marine nutrient agar, and the plates were incubated at 37 °C for 24 h. The colonies that appeared per plate of each sample were subjected to purification. The production of EPS was shown by the formation of a mucous colony, which was followed by the alcohol-based precipitation of EPS. Fresh cultures of marine isolates were applied to solid agar medium (g/L): glucose 20, CaCO_3_ 0.1, NH_4_NO_3_ 0.8, K_2_HPO_4_ 0.6, KH_2_PO_4_ 0.05, MnSO_4_.4H_2_O 0.1, yeast extract 1.0 and agar 15, and they were incubated separately at 37ºC. Mucous colony formation was examined by observing the presence of slimy mucous colonies on the plate for 3 days. Furthermore, loopful colonies were immersed in alcohol to observe the precipitation of EPSs. When a loopful of culture is mixed with pre-chilled absolute ethanol, colonies precipitate out from the solution, confirming the EPSs producer.

#### Screening for production of EPSs

Isolates were screened for the production of EPSs in a modified MY medium with 75% seawater. The medium is composed of (g/L): peptone 4.0, yeast extract 2.0, and sucrose 20.0, pH 7^57^. The medium was distributed in an Erlenmeyer flask of 250 mL, containing 50 mL of working volume. The flasks were autoclaved and inoculated using an actively growing culture and incubated at 37 °C for 3 days. The fermented broth was collected and centrifuged at 5000 rpm at 4 °C for 30 min at Sigma-Laborzentrifugen, 2K15, to remove bacteria cells. TCA (5%) was added and left overnight at 4 °C, then was centrifuged at 5000 rpm to remove protein^58^. The pH of the clear solution was adjusted to 7.0 with 0.1 M NaOH, and the crude EPS was precipitated by the addition of 4 volumes of pre-chilled absolute ethanol to the supernatant liquid; this was stored overnight at 4 °C. The sample was then centrifuged, and after decanting off the ethanol solution, the recovered pellet was re-dissolved in deionized water. The dissolved crude EPS was subjected to another precipitation step with pre-chilled absolute ethanol, followed by subsequent centrifugation and decantation of ethanol as described above. The pellet obtained was then re-dissolved in a minimum of deionized water and dialyzed three times (1000 mL × 3) against flowing tap water using dialysis tubing (Cut off MWCO 3500 Da) for 24 h. The dialyzed solution was subjected to fractional precipitated by 1, 2, 3, and 4 volumes of pre-chilled absolute ethanol. The major yield fraction obtained by 1 volume of absolute ethanol was dried under vacuum at 40 °C^59^. The UV absorption spectrum was recorded using a UV–Vis Spectrophotometer (2401PC Shimadzu, Japan) between 200 and 800 nm, to examine the existence of proteins and nucleic acids^3^. The main fraction of EPS from 1 volume ethanol (BMEPS) was assayed in-vitro against Acetylcholinesterase inhibition.

#### Identification of promising isolate

The promising isolate (S16), which produced high amounts of EPS and acetylcholine esterase inhibitor, was identified based on the biochemical, morphological, and physiological characteristics of the potential producer as determined by adopting standard methods^60,61^ The strain was confirmed with the 16S ribosomal RNA gene sequence and compared with other bacterial sequences by using NCBI BLAST. The taxonomic affiliation of the sequences was retrieved from the classifier program of the ribosomal database project^62,63^.

The genomic DNA of the isolates was extracted using the Bacterial Genomic DNA Extraction Kit. The amplification process took 2:35 total time. On a 0.8% agarose gel dyed with a DNA-safe stain, the PCR products were seen. Finally, the PCR products were sequenced, and the obtained sequence data were analyzed using the basic local alignment search tool (BLAST) software (http://www.ncbi.nlm.nih.gov/blast) against the 16S ribosomal RNA sequence database, with the mega X software (http://www.ncbi.nlm.nih.gov/mahalik) to generate the phylogenetic tree from the national center for biotechnology.

The 16S rRNA gene fragment was compared with the NCBI nucleotide database using Blastn. The following bacterial 16S rDNA from taxonomically characterized homologues were collected from the Genbank database on NCBI (http://www.ncbi.nlm.nih.gov/genbank) and was used for phylogenetic analysis.

### Characterizations of BMEPS

#### Monosaccharide, uronic acid, and sulfate composition analysis

BMEPS (50 mg) was subjected to hydrolysis with 6 N HCl for 4 h at 100 °C in a sealed tube. In a water bath at 40 °C, the HCl was removed, and (1 mL × 3) co-distilled with water^64^. Uronic acids were estimated at 525 nm using the *m-*hydroxybiphenyl procedure. Sulfate in the hydrolysate was evaluated according to the Dodgson method^65^. The hydrolysate mono-sugars were analyzed by the Agilent HPLC model 1100 series (Agilent USA)^6^.

#### UV and FT-IR spectra analysis

UV–Vis spectroscopy was conducted on the 2401PC UV–Vis spectrophotometer Shimadzu, Japan, in the wavelength range of 190–700 nm. FTIR was measured in the range of 400–4000 cm^−1^ on a Vector 22 Spectrophotometer Bucker^14^.

#### Molecular weight determination

The weight average molecular weight (*Mw*) of the BMEPS was measured using high-performance gel permeation chromatography (HP-GPC) using Agilent HPLC1100 series system according to You et al.^55^.

### Biological evaluation of polysaccharides

#### Antioxidant activities

##### Reduction of Ferric ions (Fe^3+^) power

The Fe^3+^reducing power of BMEPS was assayed according to the method of Oyaizu^66^ and was compared with BHT and Ascorbic Acid as reference materials. Successive concentrations from polysaccharide and standard materials; butylated hydroxytoluene (BHT) and Ascorbic Acid (LAA) were prepared as 100, 250, 500, 750, and 1000 µg/ml in methanol. The activity expressed as IC_50_ was calculated by log-probit analysis.

##### Ferrous ions (Fe^2+^) chelating capacity

The Fe^2+^ chelating activity of BMEPS was estimated according to the method of Dinis et al*.*^67^ and was evaluated by comparing it with two standard compounds (BHT and Ascorbic Acid at the same conditions). The percentage of inhibition of ferrozine-Fe^2+^complex formation was given by the formula:

$$ {\text{Inhibition }}\left( \% \right) = \left[ {\left( {{\text{A}}_{0} - {\text{A}}_{{1}} } \right)/{\text{ A}}_{0} } \right] \, \times { 1}00. $$

where *A*_0_ was the absorbance of the control, and *A*_1_ was the absorbance in the presence of the sample of polysaccharide and standards.

#### Free radical scavenging characters

##### DPPH radical scavenging activity

According to the method of Yamaguchi et al*.*^23^, the free radical scavenging activity of BMEPS was determined using 1,1-diphenyl-2-picryl-hydrazil (DPPH^•^). According to the following equation DPPH radical scavenging activity was calculated:

$$  {\text{DPPH}}^{ \cdot } {\text{scavenging}}\;{\text{effect}}\left( \%  \right) = 100 - \left[ {\left( {{\text{A}}_{0}  - {\text{A}}_{1} } \right)/{\text{A}}_{0} } \right) \times 100].   $$

where *A*_0_ was the absorbance of the control and *A*_1_ was the absorbance in the presence of MBES.

##### ABTS radical cation scavenging activity

Based on the method described by Miller and Rice-Evans^68^, and modification by Arnao et al.^69^, The ABTS radical cation scavenging activity of BMEPS was estimated. The ABTS radical cation scavenging activity was calculated as follows:

$$ {\text{ABTS}}\;{\text{radical}}\;{\text{cation}}\;{\text{scavenging}}\;{\text{activity}}\left( \%  \right) = \left[ {1 - \left( {{\text{A}}_{{{\text{sample}}}} /{\text{A}}_{{{\text{control}}}} } \right)} \right] \times 100.  $$

##### Lipid peroxidation-ammonium thiocyanate

The ability of BMEPS to inhibit lipid peroxidation was determined according to the method of Gülçin et al.^70^. The inhibition of lipid peroxidation in percentage was calculated by the following equation:

$$ {\text{Lipid}}\;{\text{Peroxidation}}\;{\text{Inhibition}}\left( \%  \right) = \left[ {1 - \left( {{\text{A}}_{1}  - {\text{A}}_{3} } \right)/{\text{A}}_{2}  - {\text{A}}_{4} } \right)] \times 100  $$

where A_1,_ A_2,_ A_3,_ andA_4_ was the absorbance of the sample at 1st day, 2nd, 3rd, and 4th days.

#### ROS scavenging capacity

##### Superoxide anion scavenging activity

The measurement of the superoxide anion (O^2−^) scavenging activity of BMEPS was based on the method described by Liu et al*.*^71^. O^2−^ scavenging was calculated using the following formula:

$$  {\text{The}}\;{\text{O}}_{2}^{\_} {\text{scavenging }}\%  = \left[ {\left( {{\text{A}}_{0}  - {\text{A}}_{1} } \right)/{\text{A}}_{0} } \right] \times 100.  $$

where *A*_0_ was the absorbance of the control, and *A*_1_ was the absorbance of polysaccharide or standard samples.

##### Nitric oxide radical scavenging activity

Using sodium nitroprusside (SNP), the NO^•^ radical scavenging activity of BMEPS was determined. NO^•^ generated from SNP in an aqueous solution at physiological pH to produce nitrite ions which were measured by Greiss reagent^72^.

##### Anti-inflammatory activity

The cyclooxygenase inhibition activity of BMEPS was performed according to Larsen et al.^73^ and Celecoxib was used as the standard compound.

##### Cholinesterase inhibitory effect

*Acetylcholinesterase (Ach*E*) and Butyrylcholinesterase* (*BCh*E)* inhibitory assessment.*

The enzymatic activity of BMEPS was analyzed using the method of Ingkaninan^74^. The percent inhibition was calculated using the formula:

$$ \left( {{\text{Control}}\;{\text{absorbance}} - {\text{sample}}\;{\text{absorbance}}} \right)/{\text{control}}\;{\text{absorbance}} \times 100. $$

##### Tyrosinase inhibition activity assessment

Tyrosinase inhibition assay of BMEPS was performed according to the methods of Liu et al.^75^. The anti-tyrosinase ability of BMEPS was compared to kojic acid (Sigma, St. Louis, MO, USA). The percentage of tyrosinase inhibition was calculated as follows:

$$ \left[ {\left( {{\text{A}}_{{{\text{control}}}}  - {\text{ A}}_{{{\text{sample}}}} } \right)/{\text{ A}}_{{{\text{control}}}} } \right] \times 100 $$

### Statistical analysis

The data were presented as median ± SE. *In-vitro* antioxidant data were analyzed by t-test in one-way ANOVA (n = 3 replicates) using MIB-SPSS, 25.0 software. The *P* value was less than 0.05.

## Supplementary Information

Supplementary Figure S1.

## Data Availability

All data generated or analyzed during this study are included in this published article.
