# Supplementary material for: Characterization and in-vitro Alzheimer’s properties of exopolysaccharide from Bacillus maritimus MSM1
Source: Sci Rep. 2023 Jul 14;13:11399. doi: 10.1038/s41598-023-38172-z (PMC10349148; doi:10.1038/s41598-023-38172-z)
Supplement: Supplementary file 1 — Supplementary Figure S1. [file 41598_2023_38172_MOESM1_ESM.docx]

**Supplementary Data**

**Characterization and *in-vitro* Alzheimer's Properties of Exopolysaccharide from *Bacillus maritimus* MSM1**


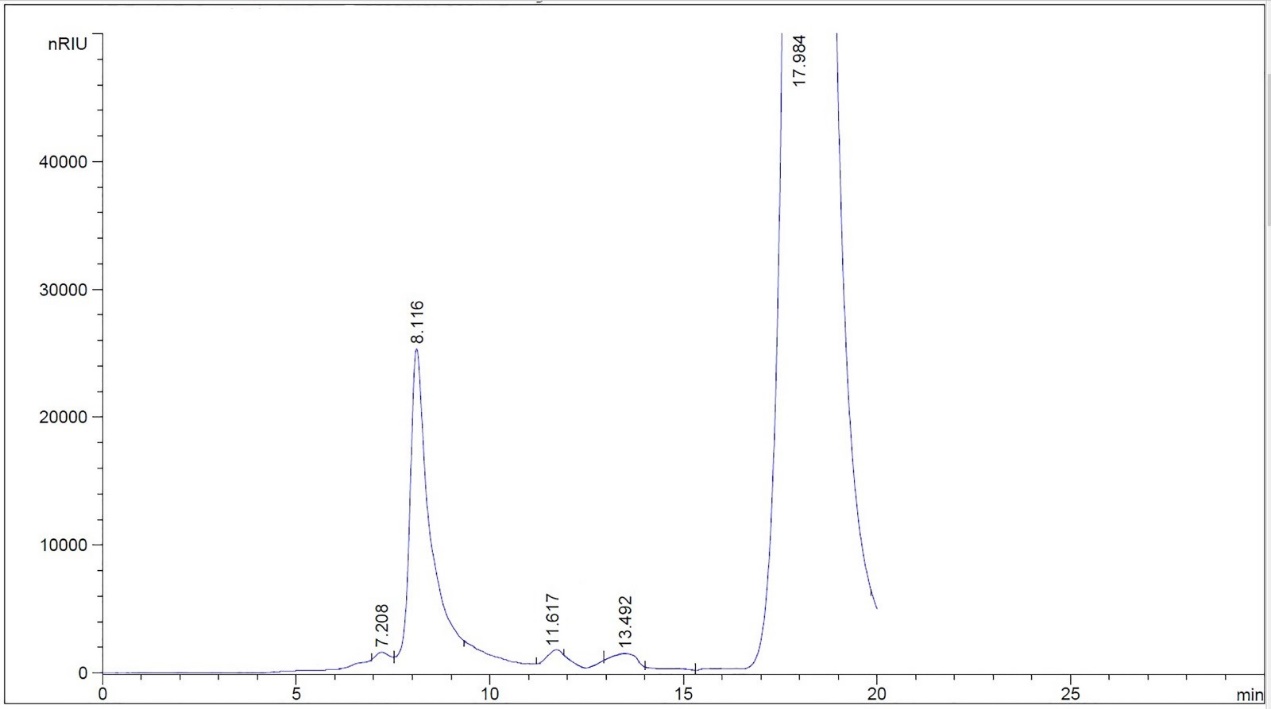


**Fig. S1.** HPLC analysis of the EPS from Bacillus maritimus MSM1
